# Heat-Treated Aramid Pulp/Silica Aerogel Composites with Improved Thermal Stability and Thermal Insulation

**DOI:** 10.3390/gels9090749

**Published:** 2023-09-14

**Authors:** Zhi Li, Kai Shen, Min Hu, Yury M. Shulga, Zhenkui Chen, Qiong Liu, Ming Li, Xiaoxu Wu

**Affiliations:** 1 School of Resources and Safety Engineering, Central South University, Changsha 410083, China; 2 Federal Research Center of Problems of Chemical Physics and Medicinal Chemistry, Russian Academy of Sciences, Academician Semenov Avenue 1, Chernogolovka, 142432 Moscow, Russia; 3 Department of Engineering Technology and Application, The Army Infantry College of PLA, Nanchang 330103, China

**Keywords:** silica aerogel, aramid pulp, heat treatment, thermal stability, thermal insulation

## Abstract

In this work, we prepared heat-treated aramid pulp/silica aerogel composites (AP/aerogels) and investigated in detail the feasibility of improving thermal stability and thermal insulation via tailored heat treatment. The microstructure and FTIR spectra reveal that AP/aerogels are formed by a physical combination of the silica aerogel matrix and aramid pulps. When the heat treatment temperature increases, the density slightly decreases and then increases to the maximum due to the significant volume shrinkage. The pyrolysis of aramid pulp and the collapse of silica skeletons occur during heat treatment; nevertheless, the typical structures of AP/aerogels do not change significantly. It is also found that both the hydrophobicity and the thermal insulation decrease with the increasing heat treatment temperature. We note that when the heat treatment is at 600 °C, the AP/aerogel still maintains a low density of 0.19 g/cm^3^ and a contact angle of 138.5°. The thermal conductivity is as low as 26.11 mW/m/K, measured using the transient hot wire method. Furthermore, the heat-treated AP/aerogels can avoid heat shock and possible thermal hazards during practical thermal insulation applications. The onset temperatures of the thermal decomposition of AP/aerogels increase from 298.8 °C for an untreated one to 414.7 °C for one treated at 600 °C, indicating that the thermal stability of AP/aerogels is improved significantly. This work provides a practical engineering approach to expand the thermal insulation applications of silica aerogel composites.

## 1. Introduction

Silica aerogels have broad applications in numerous fields, such as thermal insulation [1,2,3,4,5], catalyst carriers [6,7], aerospace [8,9] and adsorption [10,11,12,13]. However, due to the nanoporous structure and high porosity, silica aerogels exhibit high brittleness and low integrity. To improve this issue, self-reinforcement [14,15,16,17] or high-performance reinforcement materials have been widely employed to strengthen silica aerogels, e.g., polymer-reinforced [18,19] and fiber-reinforced [20,21,22] silica aerogel composites.

Aramid fibers (poly(p-phenylene terephthalamide), PPTA) have high strength, high elastic modulus, and high-temperature resistance [23,24,25]. Of these, aramid pulp, fibrillated from PPTA fibers, has higher surface activity and is easier to disperse in other substances [26,27]. Currently, it has been widely used as one of the high-performance reinforcement materials. For example, Lertwassana et al. [28] developed aramid pulp/ carbon-fiber-reinforced polybenzoxazine composites as high performance friction materials. Note that the onset temperature of the degradation of aramid pulp is up to 563 °C, while the degradation temperature at 5% weight loss of aramid-pulp-reinforced polybenzoxazine composites is only 408 °C. Li et al. successfully prepared aramid-pulp-reinforced silica aerogel composites (AP/aerogels), exhibiting monolithic shape, excellent mechanical strength and tailored thermal properties [20]. However, the thermal stability of prepared AP/aerogels primarily depends on the pure silica aerogel component (~260 °C). Once reaching this temperature, the thermal decomposition of silyl methyl groups (Si-(CH_3_)_3_) on silica aerogels occurs, even leading to open flame combustion after ignition [29,30,31]. This shows that the potential thermal hazards (thermal decomposition, fire) of AP/aerogels would seriously limit their practical thermal insulation applications.

As discussed above, the thermal stability of AP/aerogels is determined by pure silica aerogels. Adjusting the thermal stability of silica aerogels leads to the enhancement of the thermal stability of AP/aerogels. Regarding this aspect, Zhang et al. [32] demonstrated the validity of using magnesium hydroxide as a dopant to improve the flame retardancy of hydrophobic silica aerogels using facile post-doping. Yang et al. [33] enhanced the thermal stability of silica aerogel using Al_2_O_3_ atomic layer deposition.

Heat treatment is another way to adjust the thermal safety of silica aerogels, without adding any additives, which avoids changing their compositions and properties. Currently, a large number of researchers have focused on the effects of heat treatment on pure silica aerogels [34,35]. For example, Wu et al. [35] treated silica aerogels in air atmosphere, investigated the effects of heat treatment and found tailored heat treatment was feasible to reduce the flammability of silica aerogels. Lei et al. [36] proved that high-temperature treatment significantly impacted the pore structure of aerogels and improved their thermal insulation performance. Compared to oxidative atmospheres, inert atmospheres can provide a certain degree of protection for organic groups by preventing their thermal oxidation during heat treatment. In this regard, Li et al. [37] improved the thermal stability of hydrophobic silica aerogels to ~590 °C using heat treatment in an argon atmosphere at 700 °C, which greatly enhanced the thermal safety of silica aerogels. It can be seen that heat treatment is worth trying for adjusting the thermal properties of silica aerogels [38].

In this study, aramid pulp/aerogel composites (AP/aerogels) were prepared using a classical two-step, acid-base-catalyzed sol-gel process [39] and the effects of heat treatment on the microstructure, hydrophobicity, thermal stability and thermal insulation of AP/aerogels were investigated in detail. This work aims to check the feasibility of heat treatment in improving the thermal properties of AP/aerogels, and provides engineering guidance for the practical thermal insulation applications of aerogel materials.

## 2. Results and Discussion

### 2.1. Shrinkage, Density and Porosity

**Figure 1:**
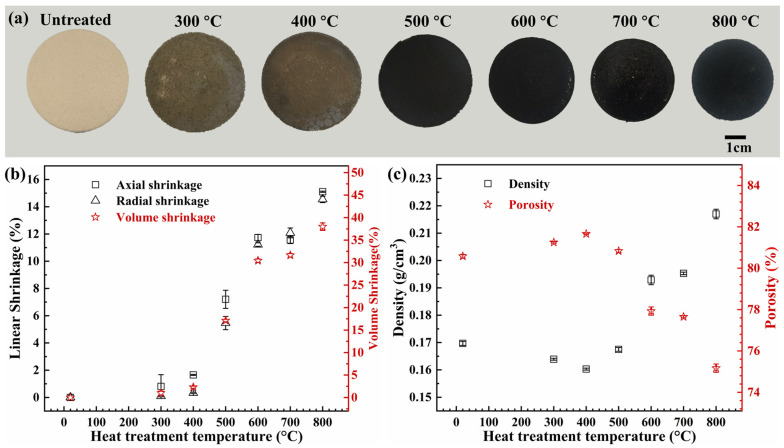
(**a**) Pictures of AP/aerogels before and after heat treatment at different temperatures, (**b**) linear and volume shrinkages, and (**c**) density and porosity of AP/aerogels under various heat treatment temperatures.

The untreated AP/aerogels, and the ones treated at various temperatures, are presented in Figure 1a. With the increase in heat treatment temperature, the AP/aerogels gradually change from grayish yellow to brown, and finally turn into black; meanwhile, their volume shrinkages become evident.

The linear and volume shrinkages of AP/aerogels at different heat treatment temperatures are presented in Figure 1b in detail. As a whole, the axial and radial shrinkages have similar tendencies, indicating the isotropic property at these two directions. They will be shown as the shrinkage of the volume overall. When the temperature does not exceed 400 °C, the volumes of the AP/aerogels do not change distinctly and the axial and radial shrinkages are kept below 2%. As the heat treatment temperature increases to 500 °C, the AP/aerogels alter from brown to black, accompanied by an axial shrinkage of 7.21% and a radial shrinkage of 5.46%. The volume shrinkage is up to 17.12%. With the heat treatment temperature further increasing, the axial and radial shrinkages rise up dramatically. These two directional shrinkages can even reach up to ~15% after heat treatment at 800 °C, with a volume shrinkage of 37.99%. In this stage, the linear and volume shrinkage are considered to be induced by the structural change in AP/aerogels. And the color transformation is related to the carbonization of organic components in the AP/aerogels.

The variations of the density and porosity of AP/aerogels can be seen in Figure 1c. When the temperature does not exceed 400 °C, the density of AP/aerogels slightly decreases from 0.17 g/cm^3^ to 0.16 g/cm^3^. The initial decreasing of the density is primarily ascribed to the reduction in mass, which is caused by the evaporation and volatilization of the residual water and organic solvents in the AP/aerogel. With the heat treatment temperature further increasing from 500 °C to 800 °C, the density rises up to 0.22 g/cm^3^ due to the significant volume shrinkage, which is mainly attributed to the damage of the porous structure of AP/aerogels. As is known, the porosity has an opposite tendency to the density, therefore it first rises up slightly and then decreases dramatically. The lowest porosity can still be maintained at 89.2%, even after heat treatment at 800 °C.

### 2.2. Microstructure

**Figure 2:**
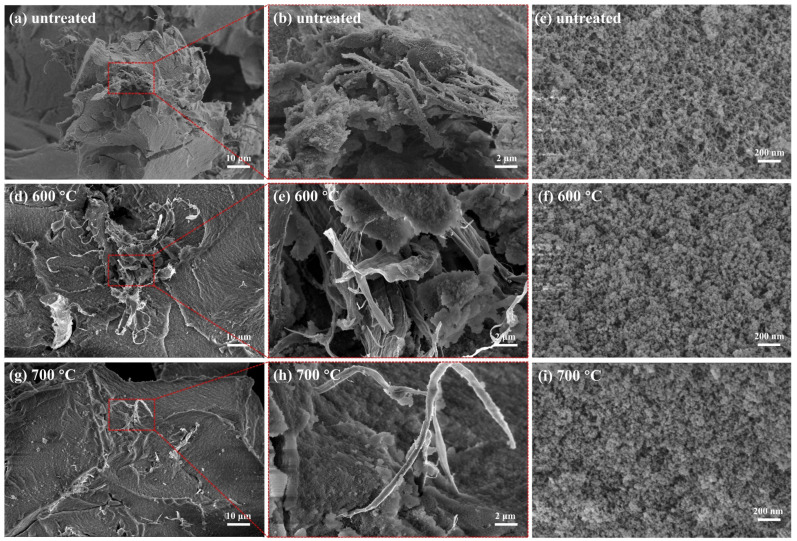
Microstructures of (**a**–**c**) the untreated AP/aerogels, (**d**–**f**) the AP/aerogels after 600 °C heat treatment and (**g**–**i**) the AP/aerogel after 700 °C heat treatment.

The microstructures of untreated and heat-treated AP/aerogels are presented in Figure 2. It can be seen that the aramid pulp is embedded in the aerogel matrix, exhibiting a random distribution in Figure 2a. The multidirectional aramid pulp acts as a supporting skeleton without causing obvious damage to the aerogel matrix, maintaining the integrity of the AP/aerogels. Figure 2b is a partial enlargement image of Figure 2a, which provides a clearer view of the bonding between the aramid pulp and the aerogel matrix. It was found that some aramid pulp is inset in the aerogel matrix and some aramid pulp stretches out of the aerogel matrix, covered by a thin layer of silica aerogel particles. Those silica aerogel particles attached to the surface of aramid pulp should be caused by electrostatic adsorption. Considering that the aramid pulp does not chemically react with substances in the preparation process of AP/aerogels, the interface binding between aramid pulp and silica aerogels is considered to be a physical combination. As shown in Figure 2c, a good three-dimensional silica network is still maintained, indicating that the introduction of aramid pulp does not destroy the nanoporous structure of silica aerogels. These structural characteristics are of great significance for maintaining the good performance of the AP/aerogels.

Furthermore, some publications have reported aramid fiber/aerogel composites [21], in which aramid fibers with a larger diameter usually cause cracks and fragmentations in the aerogel matrix. This situation can result in aerogel fragments falling off the composite, which is a common and typical issue in fiber-reinforced aerogel composites, e.g., aerogel blankets. In contrast, the highly branched aramid pulp has a higher aspect ratio for the same length, making it more conducive to interface bonding with the aerogels matrix [20,40], avoiding the separation between the aerogels and aramid pulp.

The microstructures of AP/aerogels after heat treatment at 600 °C and 700 °C are presented in Figure 2d–i. As reported, the thermal degradation of aramid pulp starts at 417.8 °C due to the heterolytic rupture of the amide bonds [20]; however, the pyrolyzed aramid pulp still can be seen inset in the aerogel matrix. Compared with those untreated AP/aerogels (Figure 2a,b), the morphologies of the heat-treated AP/aerogels (Figure 2d,e,g,h) are almost unchanged. The bonding between the aramid pulp and the aerogel matrix has remained. With regard to the aerogel matrix in AP/aerogels after heat treatment at 600 °C (Figure 2f) and 700 °C (Figure 2i), obvious skeletal collapses are observed with secondary silica particles aggregating. In spite of this, the three-dimensional nanoporous structure is still maintained. In short, except for the pyrolysis of aramid pulp and the collapses of network skeletons, the typical structures of AP/aerogels do not change significantly, which is of great importance for keeping the excellent properties of the aerogel composites.

### 2.3. Pore Structure

**Figure 3:**
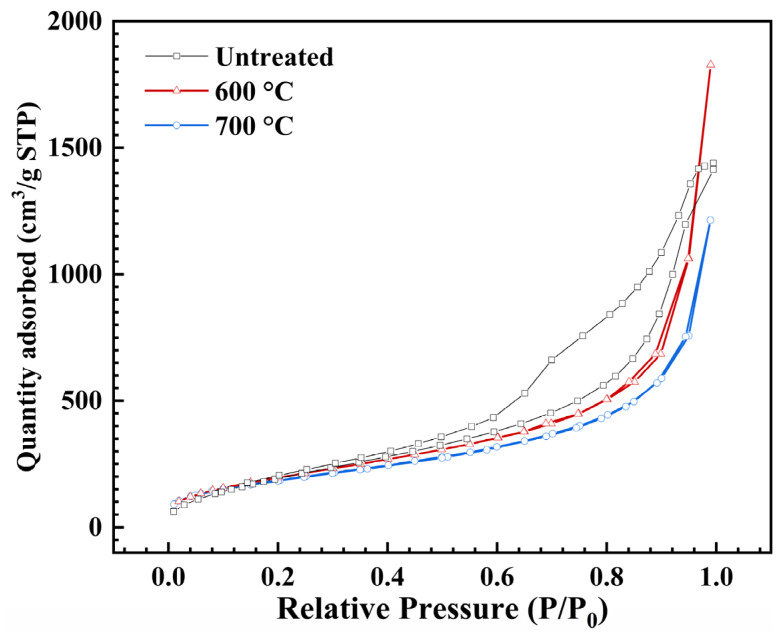
Nitrogen adsorption–desorption isotherms of the untreated AP/aerogel and ones treated at 600 °C and 700 °C.

Figure 3 shows the nitrogen adsorption–desorption isotherms of the untreated AP/aerogels and those after heat treatment at 600 °C and 700 °C, respectively. According to the classification of pore structures by IUPAC [41], all isotherms take on Type IV curves and the lathy hysteresis loops suggest the existence of mesopores in the treated AP/aerogels. The hysteresis loop of type H3 indicates the probable presence of slit-like interparticle pores. The disappearance of the saturation adsorption platform around the relative pressure of 1.0 indicates the existence of macropores and large voids in the heat-treated AP/aerogels [42].

**Figure 4:**
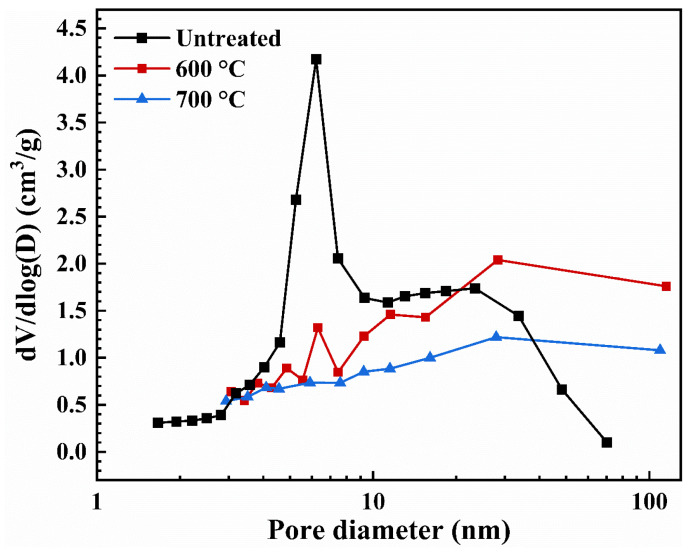
Pore size distribution of the untreated AP/aerogel and ones treated at 600 °C and 700 °C.

Furthermore, the pore size distributions of the untreated and heat-treated AP/aerogels are depicted in Figure 4. It was found that untreated AP/aerogel is composed of mostly mesopores, a few macropores and micropores, with a sharp peak ranging from 4 to 9 nm, as an indication of the most probable pore size. With the increase in heat treatment temperature, the pore size distributions change. The mesostructure of the heat-treated AP/aerogels may differ from that of the untreated ones. It is assumed that, with the aggregation of secondary silica particles, more macropores appear, which cannot be measured by N_2_ adsorption–desorption.

The pore parameters of the AP/aerogels, using the nitrogen sorption measurement and theoretical calculation, are listed in Table 1. The BET surface area of the untreated AP/aerogel is 764.8 m^2^/g. After heat treatment at 600 °C, it does not change distinctly, maintaining a value of 748.7 m^2^/g. The specific surface area is slightly larger than that of the AP/aerogel treated under 700 °C, which is 685.5 m^2^/g. With the increase in heat treatment temperature, the experimental values of pore volume and average pore size first rise up dramatically and then decrease, while the calculated theoretical pore volumes (*V_pore_*) maintain a decreasing trend. All these variations are caused by the aggregation of secondary silica particles and the collapse of the silica skeletal network during heat treatment [34,37].

Moreover, it was found that the calculated average pore diameter (*D_pore_*) of the AP/aerogel treated under 700 °C is larger than that of the AP/aerogel treated under 600 °C, which is contrary to the result obtained from the nitrogen sorption measurement. Because most macropores and voids are hard to measure during nitrogen sorption, therefore, the calculated theoretical pore volumes (*V_pore_*) are significantly larger than the experimental values. As the two treated AP/aerogels have similar *V_pore_*, a lower BET surface area leads to the AP/aerogel treated under 700 °C having a larger *D_pore_*.

### 2.4. FTIR and Hydrophobicity

**Figure 5:**
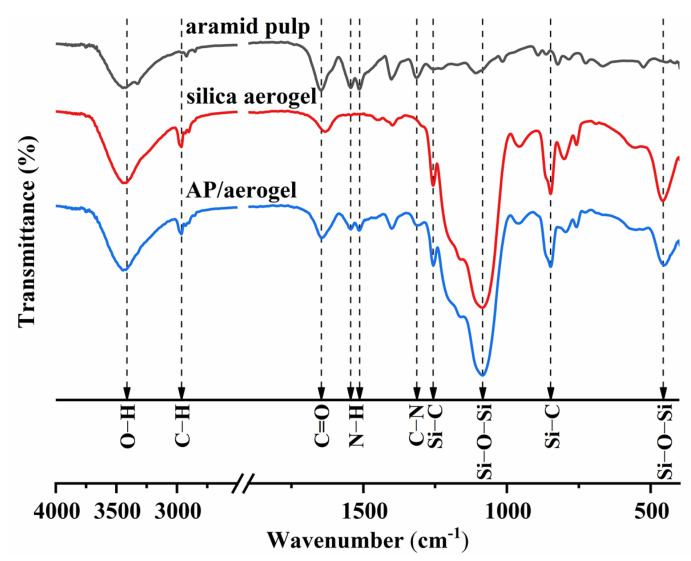
FTIR spectra of the aramid pulp, silica aerogel and AP/aerogel.

The FTIR spectra of the aramid pulp, silica aerogel and AP/aerogel are depicted in Figure 5. For the aramid pulp, the peaks at 1313 cm^−1^, 1544 cm^−1^ and 1646 cm^−1^ correspond to C–N, N–H and C=O bonds, respectively, which belong to the three characteristic bonds of amide groups (–CO–NH–) [43,44]. For the silica aerogel, the strong peak at 1085 cm^−1^ originates from the stretching vibration of Si–O–Si bonds. The bending vibration of Si–O–Si corresponds to the absorption peak at 457 cm^−1^. The absorption peaks at 1256 cm^−1^ and 848 cm^−1^ are ascribed to the stretching and bending vibrations of Si–C bonds in Si–(CH_3_)_3_ groups [45], which endow silica aerogels with good hydrophobicity. Furthermore, the broad bands around 3447 cm^−1^ are caused by OH groups and the peaks at 2963 cm^−1^ correspond to C–H bonds. For the AP/aerogel, the IR spectrum is found to overlap the silica aerogel and aramid pulp, with no chemical bonds formed. This further supports the claim that the aramid pulp only serves as a reinforcing material, and is physically combined with silica aerogels without undergoing any chemical reactions.

**Figure 6:**
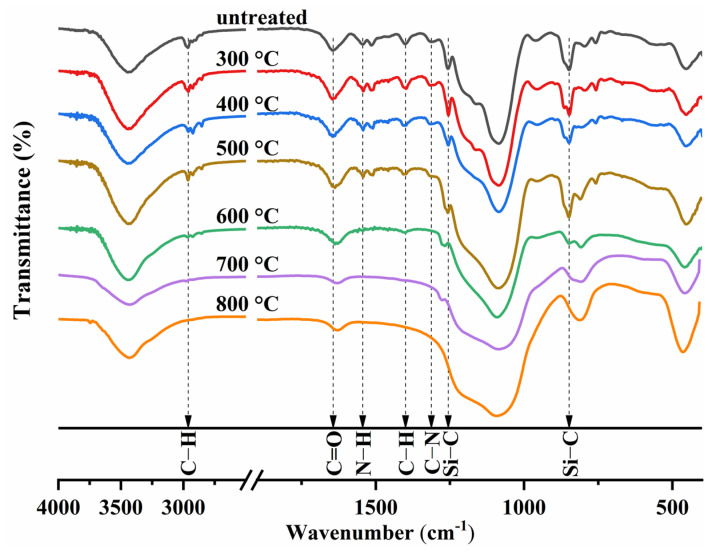
FTIR spectra of AP/aerogel after heat treatment at different temperatures.

The changes in chemical bonds and characteristic functional groups with the heat treatment temperature have been analyzed in Figure 6. Under temperatures below 500 °C, no obvious change is observed on the IR spectra of the AP/aerogel. With the heat treatment temperature reaching 600 °C, the N–H, C–N and C=O bonds disappear, implying that the amide groups are pyrolyzed between the temperature of 500 and 600 °C; meanwhile, the Si–C bonds at 1256 cm^−1^ shift to 1268 cm^−1^, as an indication of the thermal decomposition of Si–(CH_3_)_3_ groups [37,46,47]. With the heat treatment temperature further increasing, the intensities of the Si–C (1268 cm^−1^ and 848 cm^−1^) and C–H (2963 cm^−1^) bonds gradually decrease and finally disappear at the heat treatment temperature of 800 °C. These changes in chemical bonds verify the reduction in the number of methyl groups, which significantly affects the hydrophobicity of the AP/aerogel.

**Figure 7:**
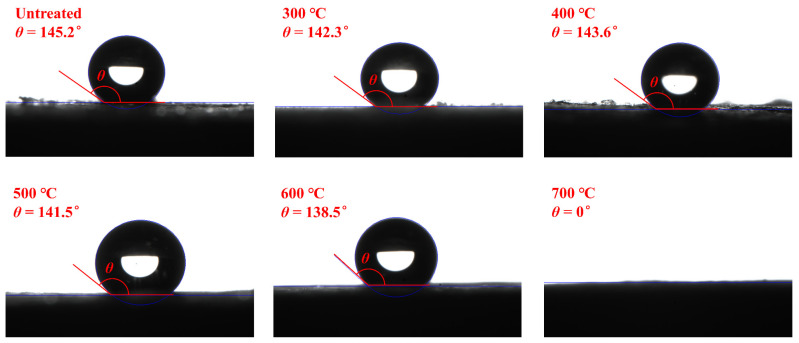
Contact angles of AP/aerogel after heat treatment at different temperatures.

The hydrophobic properties of AP/aerogels under heat treatment are investigated in Figure 7. Due to the abundant Si–(CH_3_)_3_ groups on the skeletons of silica aerogels, the AP/aerogel without heat treatment has a large contact angle (*θ*) of 145.2°, presenting good hydrophobicity. When the heat treatment temperature does not exceed 500 °C, the Si–(CH_3_)_3_ groups have no obvious change. Consequently, the contact angles of the AP/aerogels vibrate slightly and are maintained over 140°, indicating that good hydrophobicity is retained. Under heat treatment at 600 °C, the thermal decomposition of Si–(CH_3_)_3_ groups occurs, reducing the methyl groups in the AP/aerogel, which leads to the contact angle decreasing to 138.5°. As the heat treatment temperature increases to 700 °C, the AP/aerogel only has an instant contact angle of 132.1°, which will become 0° within 10 s, indicating the hydrophilicity. When the heat treatment temperature further rises to 800 °C, the AP/aerogel is also hydrophilic with a contact angle of 0°.

Based on the preceding discussion, it can be concluded that the impact on the hydrophobicity of AP/aerogels is relatively mild at heat treatment temperatures below 600 °C. These conditions continue to fulfill the demands of practical thermal insulation applications. However, beyond this temperature threshold, a shift from hydrophobicity to hydrophilicity occurs, resulting in the loss of water-resistant properties.

### 2.5. Thermal Stability

**Figure 8:**
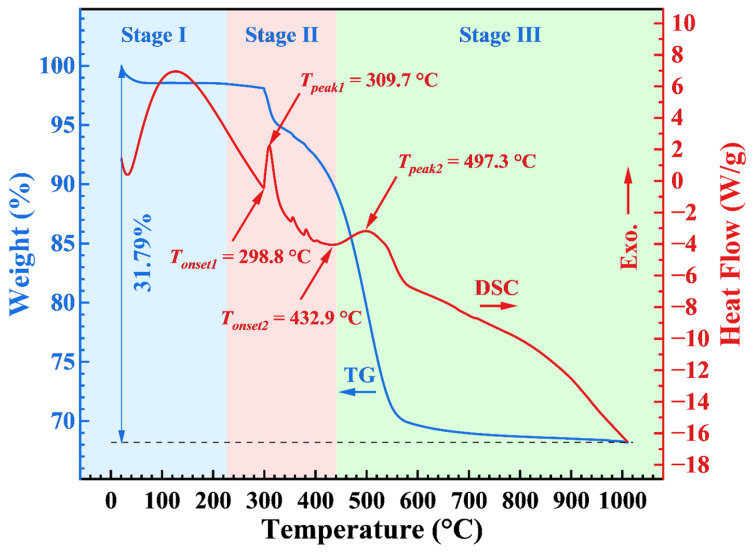
TG−DSC of the untreated AP/aerogel.

The thermal analysis of the AP/aerogel is illustrated in Figure 8. In the TG curve, it can be seen that the whole weight loss of the AP/aerogel can be divided into three stages (Stage I, II and III), with a total mass loss of 31.79%. Stage I occurs below 230 °C, and is attributed to the evaporation of water and organic solvents, such as n-hexane. The weight loss of about 8.55% that occurred in stage Ⅱ is considered to be the thermal decomposition of silyl methyl groups (Si–(CH_3_)_3_) [37]. Stage III begins at 432.9 °C, belonging to the degradation of aramid pulp [20]. In this stage, the weight loss is about 21.27%, which is even greater than the weight fraction of aramid pulp at about 18.95%. It is assumed that the thermal decomposition of residual silyl methyl groups still exists. Therein, Stage II and III have two peaks appearing in the corresponding DSC curve, respectively, indicating the exothermic characteristic of the thermal decomposition reactions.

Note that the onset temperature of the thermal decomposition of the AP/aerogel is as high as 298.8 °C, which is obviously greater than that (*T_onset_* = 248 °C and *T_peak_* = 275 °C) of pure silica aerogels [37,45], indicating that the introduction of aramid pulps can improve the thermal stability of AP/aerogels to some extent. Furthermore, the thermal stability of the AP/aerogel is still determined by the silica aerogel component, because the onset temperature of thermal degradation of aramid pulps is 432.9 °C, which is obviously higher than that of silica aerogels. Therefore, for enhancing the thermal stability of AP/aerogels, researchers should focus more on pure silica aerogels and, in this regard, heat treatment has been verified as a good strategy.

**Figure 9:**
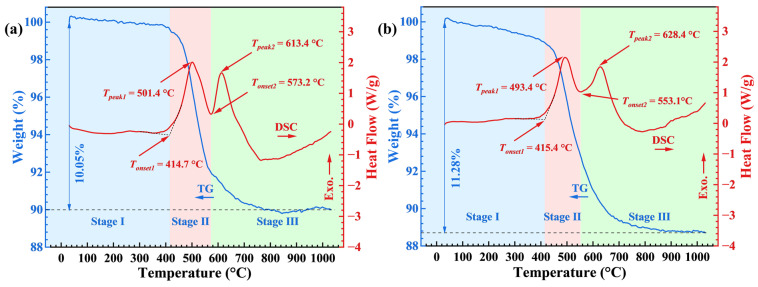
TG−DSC of AP/aerogels under heat treatment at (**a**) 600 °C and (**b**) 700 °C.

To investigate the effects of heat treatment on thermal stability, the thermal analyses of the AP/aerogels under heat treatment are presented in Figure 9. As a whole, the AP/aerogels treated at 600 °C and 700 °C have similar thermal behavior. The total weight losses of the two are both about 10%, which is obviously less than that of the untreated AP/aerogel. This is due to the thermal decomposition of some substances or functional groups during heat treatment. Similar to the untreated AP/aerogel, the whole weight losses of the treated ones can also be divided into three stages: the evaporation of residual moisture (Stage I, 0.41–1.12%), the thermal degradation of aramid pulps (Stage II, 6.08–7.69%) and the thermal oxidation of methyl groups on silica aerogels (Stage III, 1.95–4.08%). The weight loss in stage II is much bigger than the thermal decomposition of heat-treated pure silica aerogels, which has a total weight loss of ~2.8% which occurred from 523 °C [37]. This proves that it is mainly due to the thermal degradation of aramid pulp rather than silyl methyl groups. Therefore, the thermal process at Stage III has been verified. Note that, for the treated AP/aerogels, the thermal decomposition of silica aerogels occurs after that of aramid pulps, which is opposite to that of the untreated AP/aerogels. Furthermore, at Stage II and III, two distinct peaks appear accompanied by weight loss, suggesting the exothermic reaction of the corresponding thermal decomposition behavior.

The detailed thermal analysis parameters are summarized in Table 2. For the aramid pulp component, the onset temperatures of thermal decomposition are about 415 °C, which is a little lower than that of the untreated AP/aerogels. These changes should be ascribed to the damage to aramid pulps during heat treatment. For the silica aerogel component, both the onset and peak temperatures of thermal decomposition increase dramatically compared to that of the untreated AP/aerogels. Therefore, the onset temperatures improve over 250 °C and the peak temperatures increase over 300 °C, respectively. Note that they are also obviously greater than that of the heat-treated pure silica aerogels modified with trimethylsilyl groups (TSA) [37]. These improvements in the thermal stability of the silica aerogel component have been demonstrated to be caused by the evolution of the microstructure and Si-(CH_3_)_3_ groups under heat treatment [37]. From the discussion above, we prove that it is feasible to improve the thermal stability of AP/aerogels through tailored heat treatment, which is beneficial to expanding the thermal insulation application of silica aerogel materials.

### 2.6. Thermal Insulation

**Figure 10:**
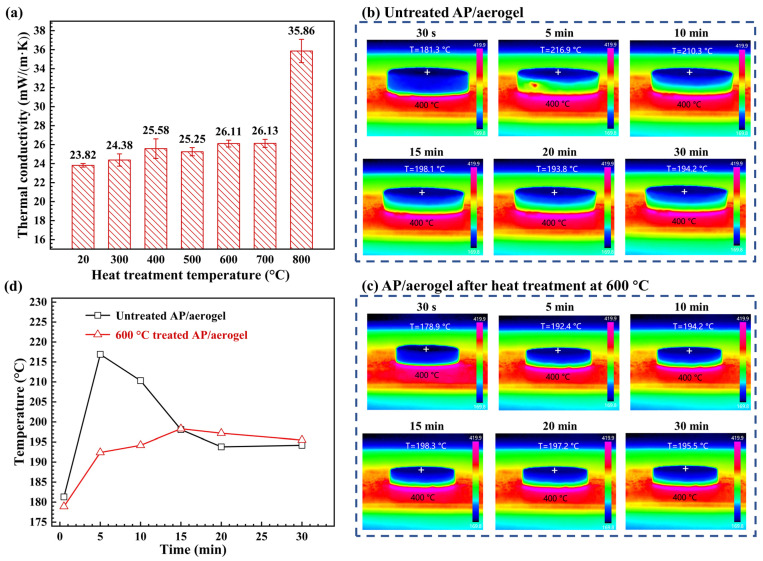
(**a**) Thermal conductivities of the AP/aerogels under different heat treatment temperatures, (**b**,**c**) infrared thermographic images and (**d**) the upper surface temperature of the AP/aerogels untreated and treated at 600 °C.

Figure 10a shows the thermal conductivities of the AP/aerogels under different heat treatment temperatures. The untreated AP/aerogel has a thermal conductivity of 23.82 mW/m/K. With the heat treatment temperature increasing to 700 °C, the thermal conductivity slightly increases to 26.13 mW/m/K, still remaining close to that of static air. Under heat treatment at 800 °C, the thermal conductivity dramatically increases to 35.86 mW/m/K, indicating the thermal insulation performance of the AP/aerogel decreases significantly. Evidently, the increase in thermal conductivity is caused by the evolution of microstructures during heat treatment.

Figure 10b,c displays the surface temperature profiles of the AP/aerogels placed on a 400 °C hot plate and their upper surface temperatures are presented in Figure 10d. For the untreated AP/aerogel, the upper surface temperature dramatically increases to the highest temperature of 216.9 °C at 5 min. Then, the temperature slowly decreases and stays at 194.2 °C at 30 min. For the AP/aerogel treated at 600 °C, the upper surface temperature increases to 192.4 °C at 5 min. Then, it further rises up to the highest temperature of 198.3 °C at 15 min; subsequently, the temperature slightly decreases and stays at 195.5 °C at 30 min.

Comparing the upper surface temperatures of the two specimens, the notable difference is that the untreated AP/aerogel has a rapid temperature rise and a distinct temperature peak within the initial minutes. When placing the untreated AP/aerogel on this 400 °C hot plate, heat transfers axially and descends gradually. It must exist somewhere that the temperature exceeds the thermal stability of silica aerogels. Once this temperature is reached, the thermal oxidation of silyl methyl groups (Si–(CH_3_)_3_) on silica skeletons occurs accompanied by heat release. The heat released by thermal decomposition is superimposed on the transferred heat, which together leads to a sharp rise in the upper surface temperature and the emergence of a temperature peak. Furthermore, this thermal decomposition behavior not only easily results in heat shock in the thermal insulation process, but may also induce fire accidents.

In summary, the AP/aerogels under heat treatment below 700 °C can still maintain low thermal conductivity without obviously impairing the thermal insulation performance. Meanwhile, the heat-treated AP/aerogels avoid heat shock and possible thermal hazards during practical thermal insulation applications. In this regard, an appropriate heat treatment is conducive to the application expansion of silica aerogel materials.

## 3. Conclusions

In this study, the effects of heat treatment on the microstructure, hydrophobicity, thermal stability and thermal insulation of aramid pulp/silica aerogel composites (AP/aerogels) were investigated in detail. A physical combination between the silica aerogels and aramid pulps is verified and the AP/aerogels undergo volume shrinkage and pyrolysis, as well as changes in the microstructure, density, hydrophobicity and thermal conductivity, during heat treatment. Under heat treatment at 600 °C, the thermal stability of the AP/aerogels is significantly improved compared to that of the untreated ones. Meanwhile, it was found that the AP/aerogels still present good thermal insulation, and avoid heat shock and possible thermal hazards during practical thermal insulation applications. This work validates the feasibility of heat treatment as an effective approach to improve the thermal stability and thermal insulation of AP/aerogels, which is beneficial for expanding the thermal insulation application of silica aerogel materials.

## 4. Materials and Methods

### 4.1. Materials

The para-aramid pulp with a length of 1.15 ± 0.05 mm was purchased from Cangzhou Zhongli New Material Technology Co., Ltd., Cangzhou, China and was produced from poly(p-phenylene terephthalamide) (PPTA) fibers like Kevlar of DuPont. The chemicals, including tetraethoxysilane (TEOS, *m*(SiO_2_) ≥ 28.4%), ethanol (EtOH, 99.7%), n-hexane (97.0%), hydrochloric acid (HCl, 36.5%) and ammonia water (NH_3_∙H_2_O, 26.5–28.5%), were purchased from Sinopharm Chemical Reagent Co., Ltd., Shanghai, China. Trimethylchlorosilane (TMCS, 98%) was bought from Shanghai Aladdin Biochemical Technology Co., Ltd., Shanghai, China. Deionized water (DI∙H_2_O) was prepared using an ultra-pure water machine (ECO-S, HHitech, Shanghai, China) and was used to prepare 0.1 M HCl (aq) and 0.5 M NH_3_·H_2_O (aq), respectively.

### 4.2. Preparation of Heat-Treated AP/Aerogels

**Figure 11:**
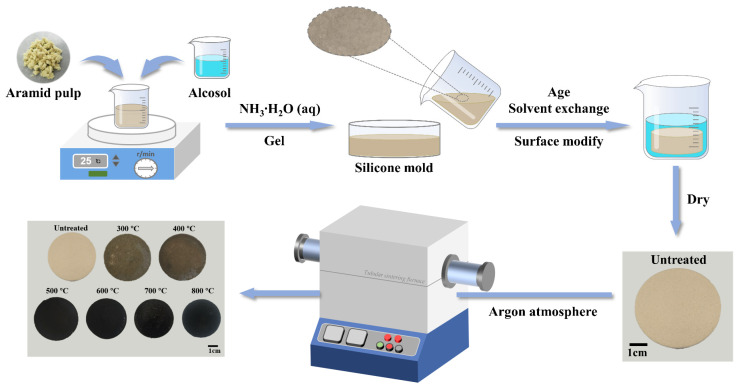
Schematic illustration of the process for synthesizing different AP/aerogels.

The AP/aerogels were firstly prepared in two steps using the classical acid-base catalyzed sol-gel process and a schematic illustration of the process is presented in Figure 11. Initially, alcosol (TEOS:EtOH:H_2_O:HCl = 1:9.6:2.16:1.6 × 10^−3^) was prepared in accordance with our previous work [21]. An amount of 0.6 g of aramid pulp was added to a measured silica sol for mechanical stirring to obtain a uniform dispersion of the pulp in the sol. Subsequently, 0.57 mL NH_3_∙H_2_O (aq) was added to the sol, which was then shaken thoroughly and poured into a cylindrical silicone mold (bottom surface diameter of 50 mm) for sealing and standing to complete gelation, which typically occurred within 20 min. After aging with ethanol, solvent exchange with n-hexane and hydrophobic surface modification with 12% TMCS/n-hexane solution, respectively, AP/aerogels were produced via ambient pressure at 60 °C and 120 °C for 4 h, respectively, to obtain the monolithic AP/aerogels. After successfully preparing AP/aerogels, they were treated under an argon atmosphere for 2 h, with temperatures of 300 °C, 400 °C, 500 °C, 600 °C, 700 °C and 800 °C, respectively. The equipment used for heat treatment is a high-temperature tubular sintering furnace (OTF-1200X-II, HF-Kejing, Hefei, China). The untreated AP/aerogels were used for comparison.

### 4.3. Methods of Characterization

The linear shrinkage was calculated as per Equation (1), and the volume shrinkage was calculated as per Equation (2):

(1) $$S_{L}=\frac{d_{0}-d}{d_{0}}\times 100\%$$

(2) $$S_{V}=\frac{V_{0}-V}{V_{0}}\times 100\%$$

in which *d*_0_ and *V*_0_ are the diameter and volume of AP/aerogel, respectively; *d* and *V* are after heat treatment.

The apparent density (*ρ*) was calculated from the mass and volume (*V*). Correspondingly, the porosity of the composite was calculated using the following formula:

(3) $$porosity=(1-\frac{\rho }{\rho_{s}})\times 100\%$$

(4) $$\frac{1}{\rho_{s}}=\frac{\omega }{\rho_{ap}}+\frac{1-\omega }{\rho_{SA}}$$

Here, *ρ_s_* is the skeleton density of AP/aerogel, *ω* is the weight fraction of aramid pulp about 18.95%, *ρ_SA_* refers to the skeleton density of aerogel about 2.20 g/cm^3^ and *ρ_a__p_* is the density of aramid pulp, usually 1.45 g/cm^3^.

The microstructures were observed using a field emission scanning electron microscope (Sigma 300, Zeiss, Oberkochen, Germany) with an accelerating voltage of 3 kV for morphology photography. To enhance the resolution for SEM observation, the specimens were sputtered with gold. Furthermore, the nanoporous structures of treated AP/aerogels were studied by N_2_ adsorption–desorption at 77 K using an automatic specific surface area and porosity analyzer (Autosorb IQ, Quantachrome, FL, USA) after a prior degassing step of 8 h at 120 °C. The specific surface areas were calculated from the nitrogen sorption using the Brunauer–Emmett–Teller (BET) method. The pore parameters were calculated from the nitrogen desorption branch using the Barrett–Joyner–Halenda (BJH) method, assuming cylindrical pores. The theoretical pore volume (*V_pore_*) and average pore diameter (*D_pore_*) of AP/aerogels were calculated as per Equations (5) and (6):

(5) $$V_{\mathrm{pore}}=\frac{1}{\rho }-\frac{1}{\rho_{s}}$$

(6) $$D_{\mathrm{pore}}=\frac{4V_{\mathrm{pore}}}{S_{BET}}$$

in which *S_BET_* is specific surface area determined using the BET method.

The chemical bonds and characteristic functional groups of AP/aerogels were analyzed using Fourier transform infrared spectroscopy (FTIR, Nicolet iS50, Thermo Fisher Scientific, Waltham, MA, USA) using the KBr compression method, and the spectral range of 4000–400 cm^−1^ was selected for the analysis. The hydrophobicity was analyzed using a water contact angle meter (ASR-7055, AISRY, Dongguan, China) with an automatic drop of 5 μL added on the surface of AP/aerogels.

The thermal analysis of the AP/aerogels was investigated using simultaneous thermogravimetry (STA 449 F3, NETZSCH, Selb, Germany) with a heating rate of 10 °C/min from room temperature to 1000 °C in air. The thermal conductivity was tested using a thermal conductivity meter (TC3000E, XIATECH, Xi’an, China) at room temperature using the transient hot wire method. The thermographic images were obtained using an infrared thermal camera (FLIR SC7300M, Wilsonville, OR, USA).

## Figures and Tables

**Table 1:** Pore parameters of the untreated AP/aerogel and ones treated at 600 °C and 700 °C.

Heat Treatment	BET Surface Area (m^2^/g)	Pore Volume ^a^ (cm^3^/g)	Average Pore Diameter ^b^ (nm)	*V_pore_* ^c^ (cm^3^/g)	*D_pore_* ^d^ (nm)
Untreated	764.8 ± 15.5	2.23	8.11	5.39	28.19
600	748.7 ± 17.6	2.73	18.47	4.68	25.00
700	685.5 ± 12.5	1.74	15.93	4.62	26.96

^a,b^ The pore volume and average pore diameter are obtained using nitrogen sorption. ^c^ *V_pore_* is calculated using the formula, *V_pore_* = 1/*ρ* − 1/*ρ_s_*. ^d^ *D_pore_* is calculated using the formula, *D_pore_* = 4*V_pore_*/*S_BET_*.

**Table 2:** The onset and peak temperatures of the thermal degradation processes.

Sample	Heat Treatment	Aramid Pulp Component		Silica Aerogel Component	
		*T_onset_* (°C)	*T_peak_* (°C)	*T_onset_* (°C)	*T_peak_* (°C)
Pure TSA [37]	Untreated	-	-	248	275
	700 °C	-	-	523	584
AP/aerogel	Untreated	432.9	497.3	298.8	309.7
	600 °C	414.7	501.4	573.2	613.4
	700 °C	415.4	493.4	553.1	628.4

## Data Availability

Not applicable.
